# Demystifying Commercial Influences on Health: Applying Systems Dynamics Methodologies to Policy Processes: Comment on "Using System Dynamics to Understand Transnational Corporate Power in Diet-Related Non-communicable Disease Prevention Policy-Making: A Case Study of South Africa"

**DOI:** 10.34172/ijhpm.9174

**Published:** 2025-07-22

**Authors:** Marsha Orgill, Lori Lake, Zakaria Belrhiti, Mumta Hargoven

**Affiliations:** ^1^The Children’s Institute, Department of Paediatrics and Child Health, Faculty of Health Sciences, University of Cape Town, Cape Town, South Africa.; ^2^International School Mohammed VI of Public Health, Mohammed VI University of Sciences and Health (UM6SS), Casablanca, Morocco.; ^3^Mohammed VI Center for Research and Innovation, Rabat, Morocco.; ^4^Global Health Policy Unit, School of Social and Political Science, University of Edinburgh, Edinburgh, UK.

**Keywords:** Systems Thinking, Complexity, Corporate Power, South Africa, Diet-Related, Non-communicable Disease

## Abstract

In their study on which we provide commentary, the authors applied a qualitative systems dynamics methodology to explore how transnational corporate power has led to policy inertia in the prevention of diet-related non-communicable disease (RD-NCD) in South Africa. This commentary explores the potential of systems thinking and causal loop diagrams to deepen understandings of – and responses to – the commercial determinants of health (CDOH). We reflect on the application of causal loop diagrams in policy processes and provide reflections that proposed strategies for change will need to take into account recent shifts in global discourse, funding streams and the balance of global power.

## Introduction

 Milson and colleagues’ case study^1^ on diet-related non-communicable disease (DR-NCD) prevention policy-making in South Africa offers an in-depth view on how to use a qualitative system dynamics method to map causal complexity. The analysis in this study uncovers what is often invisible in complex policy processes, including the forms (instrumental, structural and discursive) and mechanisms of power (ideologies, values, rules, norms, etc) that actors use to influence policy decisions or non-decisions. Importantly, this study highlights the interconnectedness of NCD drivers and patterns of policy inaction, concluding that systems mapping may support shared understanding and cooperation for the prevention of DR-NCDs.

 This study is a welcome addition to the growing literature on commercial determinants of health (CDOH), defined in a recent Lancet series as the “systems, practices and pathways through which commercial actors influence health and equity.”^2^ This definition reflects a shift from a narrow focus on “unhealthy commodities,” which feeds into industry narratives of individualised responsibility, towards broader recognition of the complex systems within which actor power, practices and policy framings are embedded. For example, Milson et al^1^ highlight how corporate actors engage in a discourse of food as a commodity (for sale) rather than as a source of nutrition and health. In the same vein, the authors note that failure to consider nutrition and health within national food security objectives undermines policy coherence and promotes policy inertia across government departments. Milsom et al^1^ thus enable a broader view of the unhealthy food environment in South Africa.

 Along with a deeper focus on systems, the CDOH literature reflects increasing interest in political economy factors, including the concepts of capitalism, neoliberalism and globalisation, in relation to commercial practices. However, Ralston et al^3^ notes that insufficient attention has been given to the institutions that link actors to these macro-level structures.^3^ Milsom et al^1^ adopt not only a political economy perspective but also shed light on how the institutions of government shape actor interactions and decisions in the policy-making process. In their findings they write that “*with increasing dominance of neoliberal beliefs, values and norms, greater importance is placed on economy-focused departments (eg, the Department of Trade and Industry) rather than health, and departmental budgets reflect this” *(p. 5). Systems thinking approaches, as applied by Milsom et al^1^ thus, can facilitate greater awareness of political economy factors that shape institutional contexts, shape perceptions of legitimacy, and in turn, actor strategies.

 While Milsom et al^1^ focus attention specifically on diet-related commercial actors and policy-making at national level, it is worth noting that the potential applications for systems thinking in CDOH research are expansive. For example, Wood et al^4^ maps the system dynamics of the ultra-processed food system on a global level, while Knai^5^ and Bertscher et al^6^ explore interconnections across different health harming industries (alcohol, tobacco, and ultra-processed foods). Given the urgency of the climate crisis,^7^ systems mapping may also be a powerful tool to explore the externalised environmental harms of corporate actors, and in so doing extend our thinking about corporate harms beyond the downstream burden of disease.

 The remainder of this commentary explores key findings of Milsom et al,^1^ discussing the systems thinking approach and reflecting on implications for public health policy and research.

 Given the complex causality of social change in health systems, applying a systems lens to understanding problems and solutions is not an easy task. Fortunately there are a growing set of tools and methodologies^8-10^ that can help us in teasing out interconnected issues within system structures, understanding feedback mechanisms, identifying leverage points, analysing dynamic behaviours, using heuristics to propose solutions, and developing simulations to test policy interventions.^8,11^ These tools enable researchers to bring together a range of perspectives through illustrative mental models that help to shape systemic understandings of problems and solutions.^11^

 Milsom et al^1^ use causal loop diagrams to illustrate why DR-NCD prevention policy-making in South Africa has stalled, despite a rising prevalence of obesity and NCDs. These causal loop diagrams were validated by the research participants, and the analysis was enriched by data from a realist review conducted by the authors.

 Key reinforcing loops, that maintain or strengthen the status quo of corporate power, include the food industry’s productive power and legitimacy in the sector, the use of lobbying and manufacturing doubt to influence agenda-setting, and the framing of food as a commodity, rather than as a source of nutrition and health. Conversely, balancing loops, which can be understood as key leverage points that shift the system in a different direction, include evidence-informed practise and policy-making, international instruments and policy norms, creating awareness of industry tactics, civil society pressure, and nutrition network mobilisation.

 Through these diagrams, Milsom and colleagues^1^ also draw attention to the institutional mechanisms that enable industry influence. For example, in South Africa, this includes the constitutional imperative to include all issue relevant stakeholders in policy-making. The authors therefore advocate the adoption of an International Framework Convention on Food Systems which could be leveraged to protect national public health policy-making from commercial interests. Problem system structure mapping thus allows us to think generatively in the process – to not only observe the embeddedness of corporate power in policy processes, but also to consider strategies that may help to contain corporate power.

 However, as recognised by Milsom et al,^1^ systems are in a state of flux and subject to internal pressures as well as shifting global political and economic forces. Changing contexts may therefore impact the dynamics within a system, as experienced during the COVID-19 crisis.^12^

 The world we inhabit in 2025 is fundamentally different to the one that the authors encountered in 2023. We have seen a dismantling of health and environmental regulatory bodies in the United States,^13^ and the defunding of the World Health Organization (WHO), United Nations (UN) and other global institutions designed to uphold public health and the human rights order.^14,15^ Within this context, establishing a robust International Framework Convention on Food Systems may prove particularly challenging. Indeed, in relation to recent UN plastics treaty negotiations, Ralston et al^16^ highlights a “democratic deficit” through which the perspectives of food and beverage corporations, which depend on single-use plastics are favoured, while civil society perspectives are marginalised.

 Complexities around the use of evidence in policy-making are also likely to be exacerbated in today’s post-Trumpian world, where science itself is under threat, and what counts as ‘truth’ has become increasingly partisan.^17^ Recent research funding cuts in LMIC countries, including South Africa, may limit the ability of scientists and civil society to exert pressure for evidence informed or evidence based policy-making.^18,19^ This highlights a need to reassess and recalibrate our strategies and alliances—both locally and globally—so that we can bolster South-South and North-South solidarity to defend public and planetary health. This could for example include regional coalitions for the advocacy of food governance and/or shared surveillance mechanisms.

 Qualitative causal loop diagrams are thus useful in depicting interrelationships and to identify possible leverage points for system change. However, the insights derived from causal loop diagrams are closely tied to the context at the time of the study and are not well suited for anticipating future behaviours of actors particularly.^8^ Fortunately, there are complementary methodological toolkits that can usefully be applied for making assumptions about future systems’ behaviours such as Social Network Analysis and quantitative simulation models. These methods allow a move from static description to dynamic adaptation and thorough understanding of how political economic macro factors interact with actual (and future) contexts and changing interests of policy actors.

## Implications for Addressing CDOH

 The authors remind us that complexity must be considered when trying to understand the intersections of corporate power and institutions within policy processes. Corporate power is not limited to one action or strategy, therefore solutions should not be too limited in scope. For example, a sugar tax may indeed reduce sugar consumption, but it may do little to reduce industry’s profits, nor their power and policy influence.^1^ An additional example in South Africa includes industry exploitation of grey areas in marketing regulations on breastmilk substitutes,^20^ using harder-to-regulate strategies such as online adverts, mother and baby clubs and care lines.^21,22^

 This highlights the importance of both policy content and effective regulation and enforcement. Building the capacity of the state to monitor compliance and respond to violations is essential. We argue that a set of indicators to monitor system level influences (eg, lobbying intensity) should be developed and embedded in routine monitoring systems to enable evaluation and adaptive learning that can inform future government strategy. But this needs to be coupled with the efforts of an informed and active citizenry. Education is key to counter industry narratives, so that policy-makers, health professionals and the general public are more aware of the drivers and full costs of DR-NCDs and better equipped to champion human and planetary health. Milsom et al recognize this element as part of the balancing loop, “nutrition network mobilisation.”

 We would suggest that this should include education on conflicts of interest, including the ways in which corporate social responsibility initiatives, funding, sponsorship and other ‘gifts’ from, for example the food industry, may compromise the integrity of health professionals, researchers and government officials and undermine public confidence and trust. Further insight could be generated specifically on the ways in which policy actors may move seamlessly between government, to civil society and industry appointments. As an additional balancing loop, we therefore recommend that conflict of interest policies, procedures and processes at both individual and institutional levels should be put in place to govern joint ventures.^23^

## Conclusion

 The Milsom et al^1^ study shows the power of causal loop diagrams as a visualisation tool for generating new insights and building shared mental models about systems interrelationships and system boundaries. Systems mapping as a methodology enables stakeholders engaged in different parts of the system, to conceptualise the world differently and to visualise the web of inter-connections that extend beyond their domain and connect them with the broader system. And in the process to identify potential allies and points of leverage that may have previously been out of sight. This is powerful knowledge, that needs to be leveraged by bureaucrats and policy-makers to drive change. We argue that developing the skills of policy-makers to do systems mapping themselves, would further institutionalise systems thinking in policy processes.

 While knowledge is powerful to enable sensemaking, we also need parliamentarians, policy-makers and senior bureaucrats who can take action and engage in systems leadership, that is the ability to work across individual, team, organisation and network levels to manage actor power and lead change in policy-making now and in the future.^24^ At the institutional level, given the inter-sectoral nature of DR-NCD prevention policy-making in South Africa, practical programme interventions, such as establishing formal procedural arrangements, to strengthen collaborative governance and intersectoral coordination mechanisms could also enable action and change.^25^

## Ethical issues

 Not applicable.

## Conflicts of interest

 Authors declare that they have no conflicts of interest.
